# Grace Under Pressure: a mixed methods impact assessment of a verbatim theatre intervention to improve healthcare workplace culture

**DOI:** 10.1186/s12913-024-10961-w

**Published:** 2024-04-16

**Authors:** Claire Hooker, Aspasia Karageorge, Karen M. Scott, Renee Lim, Louise Nash

**Affiliations:** 1 Sydney Health Ethics, School of Public Health, University of Sydney, Camperdown, NSW 2006 Australia; 2 Brain and Mind Centre, Faculty of Medicine and Health, University of Sydney, Camperdown, NSW 2006 Australia; 3 Specialty of Child and Adolescent Health, Faculty of Medicine and Health, University of Sydney, Camperdown, NSW 2006 Australia; 4 The Pam McLean Centre, Faculty of Medicine and Health, The University of Sydney, St Leonards, NSW 2064 Australia; 5 Brain and Mind Centre, Faculty of Medicine and Health, University of Sydney, Camperdown, NSW 2006 Australia

**Keywords:** Healthcare workforce retention, Psychosocial risk, Verbatim theatre, Healthcare workplace culture, Arts and health, Incivility, Bullying, Harassment

## Abstract

**Background:**

Healthcare workplace mistreatment has been documented globally. Poor workplace behaviour, ranging from incivility to bullying and harassment, is common in healthcare, and contributes significantly to adverse events in healthcare, poor mental health among healthcare workers, and to attrition in the healthcare workforce, particularly in junior years. Poor workplace behaviour is often normalised, and is difficult to address. Verbatim theatre, a form of research informed theatre in which plays are created from informants’ exact words only, is particularly suited to facilitating workplace culture change by raising awareness about issues that are difficult to discuss. The objective of this study was to assess the impact of the verbatim theatre play ‘Grace Under Pressure’ on workplace culture in NSW hospitals.

**Methods:**

The intervention was conducted in 13 hospitals from 8 Local Health Districts (LHDs) in NSW, Australia, in October and November 2019, with aggregated impact across all sites measured by a bespoke survey (‘Pam McLean Centre (PMC) survey’) at the conclusion of the intervention. This study was conducted in 3 Local Health Districts (one urban, one regional, one remote), with data collection conducted in November–December 2019 and December 2020. The study design was a mixed methods assessment of the play’s impact using (1) validated baseline measures of psychosocial risk, analysed descriptively, (2) overall findings from the PMC survey above, analysed descriptively, (3) interviews conducted within a month of the intervention, analysed thematically and (4) interviews conducted one year later, analysed thematically.

**Results:**

Half (51.5%) of the respondents (*n* = 149) to the baseline survey had scores indicating high risk of job strain and depressive symptoms. Of 478 respondents to the PMC survey (response rate 57%), 93% found the play important, 92% recommended others see the play, 89% considered that it stimulated thinking about workplace behaviour, and 85% that it made discussing these issues easier. Thematic analysis of interviews within one month (*n* = 21) showed that the play raised awareness about poor workplace behaviour and motivated behaviour change. Interviews conducted one year later (*n* = 6) attributed improved workplace culture to the intervention due to improved awareness, discussion and capacity to respond to challenging issues.

**Conclusions:**

Verbatim theatre is effective in raising awareness about difficult workplace behaviour in ways that motivate behaviour change, and hence can be effective in catalysing real improvements in healthcare workplace culture. Creative approaches are recommended for addressing similarly complex challenges in healthcare workforce retention.

**Supplementary Information:**

The online version contains supplementary material available at 10.1186/s12913-024-10961-w.

## Background

Healthcare workforce shortages and retention of workers are urgent issues, particularly in the wake of the Covid-19 pandemic [1]. One significant contributor to attrition among healthcare staff is experiencing poor workplace behaviour from colleagues, which ranges from incivility (offensive, intimidating, or insulting behaviours) to bullying and harassment [19]. Poor healthcare workplace behaviour has been documented globally [2, 3], and affects both medicine and nursing at varying rates; for example, recent reviews identified average prevalence among nurses at 26% and 66.9% (see [2, 4, 5]). Junior staff, women and minorities are more likely to be victims [6, 7]. ‘Teaching by humiliation’ and other forms of mistreatment are experienced or witnessed by a majority of medical and nursing students [4, 8–11].

Poor healthcare workplace behaviour can cause extensive harm in victims, including long lasting burnout, psychological distress, intentions to quit the profession, reduced clinical ability, reduced capacity to learn and reduced career progression [6, 12]. Such behaviour can impact on the quality of care and patient safety as a result of impaired concentration, inhibited communication and delays or errors in care [5, 13]. Positively, there is evidence suggesting that the converse is true, and that improvements in healthcare workplace culture will mitigate burnout [14] and improve cost-effectiveness and patient outcomes [6].

Although poor healthcare workplace behaviour is now widely researched, there have been few signs of improvement [4, 7, 15, 16]. Often, the problem has not even been disclosed, either to colleagues or managers. Healthcare workplace behaviour often includes attacks on the victim’s professional identity. Feelings of shame [17] (common in victims of abuse) have often rendered the issue a ‘silent epidemic’ [18]. Institutional policies have often had little impact, in part because victims fear that a complaint may negatively impact their career prospects [19]. A cycle in which juniors internalise such behaviours during training and eventually reproduce them, is in place [7, 20].

The creative arts have affordances particularly suited to intervene in poor workplace culture and halt self-perpetuating cycles. ‘Verbatim’ theatre is a form of ‘documentary’ or research-informed theatre. Verbatim theatre plays must be composed using only the exact words of real informants (usually provided in interview), who have lived experience of an issue of interest. Verbatim theatre was explicitly devised to raise awareness of discomfiting issues, especially the experiences of stigmatised social groups, from sex workers to refugees to sufferers of alopecia or those recovering from stroke. Verbatim theatre has been particularly valued for disrupting ‘social laryngitis’, that is, the perception that an issue is ‘unspeakable’ due to shame, stigma, fear or disempowered circumstances [21–23] – something that victims of bullying and harassment in healthcare work often experience [5, 19]. Multiple ‘active ingredients’ [24], including fidelity to real experience, providing witness to distress, capacity to represent complexity, and humour, mean that verbatim theatre works can generate experiential and moral learning [25, 26].

### Rationale: assessing the impact of a creative intervention

Teaching by humiliation, poor workplace culture and bullying and harassment are unfortunately prevalent in the Australian healthcare industry [27–29] and produce high rates of burnout and mental ill health among the Australian healthcare workforce [30].

The verbatim theatre play *Grace Under Pressure* (Williams and Dwyer, 2017) was created in 2017 from interviews with 30 doctors and nurses across all career stages (including students) and representing a range of healthcare roles [31], to explore training and workplace challenges in healthcare. Topics represented in the play included burnout, excessive workloads, poor work/life balance, inappropriate levels of responsibility, poor workplace behaviour ranging from incivility to bullying, sexual harassment and sexism, interprofessional hierarchy, accidents and suicide. The play also portrayed humour, collegiality, joy at work, and care. *Grace Under Pressure* was well reviewed during its mainstage debut in Sydney, Australia, and was subsequently adapted by the Pam McLean Centre (PMC) and performed in a range of healthcare workplace and educational settings, as well as achieving a national mainstage tour in 2020 and 2021. In 2019, NSW Health, the Health Ministry in the state of New South Wales, supported the play as a healthcare workplace culture intervention, consisting of a performance followed by workshops in multiple hospitals across NSW.

### Aim

Our aim was to assess the impact of this intervention. Our research questions were: (1) Did the play raise awareness about poor healthcare workplace behaviour? (2) Did the play create intentions to improve healthcare workplace culture? (3) Did the play contribute to generating actual improvements in healthcare workplace culture?

## Methods

### Intervention and research settings

The Grace Under Pressure play and workshop (‘the intervention’) was offered by PMC; NSW Health; and theatre company Alternative Facts. The intervention was conducted in November 2019 in 13 hospitals (4 metropolitan hospitals, 7 mid sized regional hospitals and 2 remote hospitals), located in 8 Local Health Districts (LHDs) across NSW, where there are 15 LHDs in total. All hospitals and LHDs who received the intervention responded to one component of impact research, a bespoke survey issued by PMC. Our additional impact research (baseline measures and qualitative interviews) was focused in 3 LHDs that typify health service contexts across the state. LHD #1 is a busy metropolitan LHD; the intervention was conducted in two hospitals. LHD #2 is a regional LHD and held the intervention in one hospital. LHD #3 is a remote LHD; the intervention was conducted in two hospitals. That is, our research assessed impacts of performances in 5 hospitals located in 3 LHDs.

### Study design

**Fig. 1:**
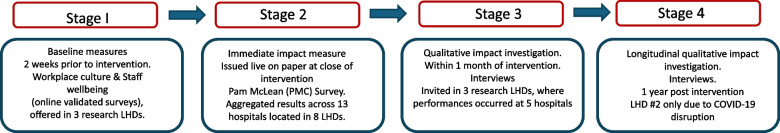
Research stages, times and methods

This research employed an exploratory mixed-methods design comprising four stages, as shown in Fig. 1.

### Variations and COVID-19 disruption

Local resource constraints and communications systems led to each LHD undertaking recruitment differently, including prioritising invitations to doctors and medical students at some sites. This resulted in an unclear total of invitations to participate in the intervention and in the research, and lower research participation in LHD#3. Post-play workshop content (delivered by PMC) was tailored to each LHD, and generally involved discussions of issues raised by the play. Due to disruption by COVID-19, only LHD #2 completed Stage 4.

### Research activities

#### Stage 1 (pre intervention baseline)

Stage 1 comprised the validated 4-item Psychosocial Safety Climate survey (PSC-4)14 [32] and the validated 9-item health practitioner wellbeing index [33]. A score >  = 3 in the Wellbeing Index places the respondent at greater risk for reporting a medical error and experiencing burnout, fatigue, suicidal ideation and/or lower quality of life [32]. In the Psychosocial Climate index, scores 41–37 indicate moderate, and scores <  = 37 indicate high, risk of job strain and symptoms of depression.

### Stage 2 (close-of-intervention)

All intervention participants across all intervention sites (13 hospitals) were invited to complete the bespoke Pam McLean Centre (‘PMC’) survey (used to evaluate PMC activities), on paper, at close of intervention, while they were still in the room. This survey contained 16 items, comprising 1 consent item, 4 demographic items, 5 Likert-scale items measuring responses to the play, 5 Likert-scale items measuring experiences of workplace misconduct and personal resilience, and 5 free-text items that explored respondent perceptions of the play and of workplace culture. We report on the aggregated data set provided by PMC here using 3 categories, Strongly Agree/Agree, Neutral and Disagree/Strongly Disagree, to provide at-a-glance understanding of the outcomes without compromise of validity [34, 35].

### Stage 3 (post intervention)

All registered audience members at LHDs #1, #2 and #3 were invited by email or at the play, to participate in a semi-structured single or group interview within one month of the intervention (supplementary materials), with attendance at the play the only inclusion criterion applied. The interview guide was developed by the research team to address the research questions. All interviews were recorded and professionally transcribed.

### Stage 4 (One year follow up)

Due to COVID-19 disruptions, only LHD #2 was able to participate in Stage 4. All LHD #2 interviewees from Stage 3 were invited to participate in a structured interview in December 2020 to explore the play’s impact at one year. The interview guide was adapted from that used in Stage 3. Interviews were recorded and professionally transcribed.

Human Research Ethics approval was granted by the University of Sydney Human Research Ethics Committee for the Phase I pre-intervention survey, and the interviews and focus groups. The Royal North Shore Hospital Human Research Ethics Committee granted Ethics approval for the PMC Survey.

### Data analysis

Survey data was analysed using simple descriptive statistics.

Interview data was analysed thematically using NVivo. The team extracted relevant key words and concepts from immersive reading, after which AK and CH constructed latent themes [36] designed to answer our research questions. Extensive reflexive discussions were used to check and develop themes and sub themes.

## Results

### Stage 1: Baseline measures of wellbeing

#### Psychosocial climate survey and wellbeing index

There were 162 respondents to the survey across 3 LHDs. Females comprised 80% (128/162) of the sample. 50% (80/159) had staff reporting to them or under their supervision and 57% (90/159) reported they made decisions that impacted on the workings of the hospital. The respondent’s main role in their LHD was: clinician 52% (83/159), administrator 21% (33/159), educator 10% (16/159), student 2% (3/159) and other 15% (24/159). For clinicians (including students), this divided further into: allied health 26% (28/108); nursing 24% (26/108); medical practitioner 42% (45/108) and other 8% (9/108).

Sixty percent of respondents scored as being at high risk of burnout, mental illness, and reporting a medical error (Table 1).

**Table 1:** Psychosocial climate survey and wellbeing index survey results by Local Health District (LHD)*

	**Wellbeing Index**^**§**^	**Psychosocial Climate Survey**^**§§**^
*LHD*	*Health professional wellbeing cut off* > = 3* (n)	Proportion scoring at high risk on PSC**(n)
LHD#1	45% (48/107)	71% (76/107)
LHD#2	37% (11/30)	31% (9/29)
LHD#3	46% (6/13)	38% (5/13)
Total	43% (65/150)	60% (90/149)

Response rate not available as the extent of distribution of this survey via LHD communication channels was not known

### Stage 2: close-of-intervention PMC Survey responses

A total of 852 people attended the play across all (13 hospital) sites in NSW. Four hundred and eighty eight (response rate 57%) responded to the PMC survey issued at the conclusion of post-play discussions or workshops. After demographic information was extracted, data for those who completed less than 75% of survey questions were excluded (*n* = 7), and descriptive statistics were used to analyse the remaining 478 responses (response rate of 55%).

The audience who attended the play was a heterogenous group of clinicians across medicine, nursing and allied health (and including dentistry, pharmacy), alongside staff with management, human resources, finance, administration, risk management, health informatics, community health and pastoral care roles (‘Other’ in Table 2).

**Table 2:** Demographic data concerning participants who attended the play

	**Pam McLean Centre Survey Demographics**	
**Survey questions**		**Total (*n* = 478; 13 sites)**
Age:	under 25 years	41 (8.6)
	26 to 35 years	106 (22.4)
	36 to 45 years	98 (20.7)
	46 to 55 years	120 (25.3)
	over 55 years	109 (23.0)
What is your gender?	Female	362 (76.2)
	Male	112 (23.6)
	Prefer not to answer	1 (0.2)
What is your discipline:	Medical	137 (28.7)
	Nursing	132 (27.7)
	Allied Health	87 (18.2)
	Other (please specify)	121 (25.4)
Length of service in years:	Mean Percentile 25 Percentile 75 Standard Deviation	17 5 27 13

The PMC Survey results showed that this heterogenous audience of healthcare staff valued the play highly (93%) (Table 3). An overwhelming majority indicated that they would recommend that colleagues, family, friends and members of the public saw the play (92%). They considered that the play stimulated their thinking about workplace behaviour (89%) and agreed that seeing the play made discussing these issues easier (85%).

**Table 3:** Participant evaluation of *Grace Under Pressure*

	Response, n (%) *		
*Item*	Strongly Agree / Agree	Neutral	Strongly Disagree / Disagree
The play Grace Under Pressure is important because it acknowledges that some people have bad experiences in healthcare workplaces (*n* = 464)	432 (93)	7 (1)	25 (5)
Overall I would recommend that healthcare workers and their friends and family go and see Grace Under Pressure (*n* = 455)	420 (92)	28 (6)	7 (1)
Seeing the play makes talking about workplace culture issues easier (*n* = 455)	388 (85)	49 (11)	18 (4)
I would like members of the public to see Grace Under Pressure or similar theatre works (*n* = 453)	385 (85)	60 (13)	8 (2)
The play stimulated my thinking about workplace culture issues (*n* = 451)	401 (89)	31 (7)	19 (4)

^*^Results presented as agree/neutral/disagree for ease of reporting

Survey responses demonstrated that the play authentically represented real healthcare work experiences. Most participants had witnessed or experienced situations similar (66%, *n* = 310) or somewhat similar (24%, *n* = 112) to those in the play. Only 11% (*n* = 50) did not relate to play content. Respondents were unlikely to disclose experiences of poor workplace behaviour to supervisors or hospital management, but would disclose such experiences to peers (see Table 4).

**Table 4:** Participant disclosure of negative workplace experiences

	Response, n (%)		
*Item*	Always or Often	Sometimes	Rarely or Not At All
If you have encountered these types of experiences, to what extent would you share them with peers? (*n* = 460)	214 (47)	170 (37)	76 (17)
If you have encountered these types of experiences, to what extent would you share them with seniors/supervisors? (*n* = 460)	97 (21)	148 (32)	212 (46)
If you have encountered these types of experiences, to what extent would you share them with hospital management/administration? (*n* = 452)	66 (15)	108 (24)	278 (62)

^*^Results presented as agree/neutral/disagree for ease of reporting

Free-text responses to the question ‘What are three things that might make you consider leaving your job?’ also confirmed the negative impact of poor workplace culture, with the top 5 answers by frequency being; Lack of support/appreciation, Too much work, Bullying, Stress, and Change.

### Stage 3: interviews (within 1 month after the play)

Six interviews (2 group (5 participants), 4 single) were conducted at LHD #1, 8 single interviews were conducted at LHD#2, and 3 single interviews were conducted at LHD #3 within 1 month after the performances. These 20 interviews included 16 females and 5 males; 3 junior doctors (Junior Medical Officer or Resident Medical Officer); 7 senior physicians; 6 nurses; 2 allied health; and 3 staff from Human Resources. Some held or had held leadership roles.

Respondents were emphatic that the play was highly valued as an important means of raising awareness of, insight into, and discussion about, healthcare workplace pressures, which they perceived as faithfully represented. Respondents commented on a range of changes they were motivated to undertake after seeing the play, and one year later, considered that the play had played a significant role in facilitating real changes in workplace culture.

One over-arching theme for each research question was constructed through latent thematic analysis. In Stage 3, these themes were ‘raising awareness’ and ‘intentions for change’. Each theme was constructed from inter-related sub themes which were derived inductively. These are briefly described below with illustrative examples.

### Raising awareness

This theme was composed of seven sub themes: (1) ‘recognition’, in which respondents recognised their own experiences in the play; (2) ‘need for raising awareness’, in which respondents commented on the need for, and value of, making the play’s issues more widely known; (3) ‘raising awareness in managers’, in which respondents made claims about the importance of managers becoming more aware of the issues raised by the play; (4) ‘already aware’, in which respondents spoke of their knowledge and experience of the issues depicted (sometimes connected with the sub-theme ‘recognition’); (5) awareness of what is working, in which respondents identified models of positive workplace culture; (6) ‘gaining insight’, in which respondents recounted how the play increased their understanding of the phenomena portrayed in it; and (7) ‘recommending the play’, which captured comments from participants who considered seeing the play valuable, usually because of one or more of the other six sub themes. Brief examples of each sub theme are provided below.

Many respondents commented that the play accurately reflected their own experiences, for example:

So yeah I felt quite overwhelmed, I think part of it was just I could relate to quite a lot of the scenarios, both from a clinical perspective but also the sense of pressure about performance and being things to all people and all those things. (Manager, male).

The perception that the play authentically represented real experiences of workplace pressure and misconduct led respondents to comment on their perceptions of issues of burnout, bullying, harassment, incivility and mental ill health at work. Some talked about how important it is to increase awareness of the issues, including the detail of how they present in the workplace:

I think the more people that are aware of what goes on behind the scenes the—because that's really the only way to effect behavioural change; to not just—I guess not just say oh people get bullied and bad things happen. But just say this is the actual breakdown of what people are experiencing and it's not appropriate (Registered Medical Officer, male).

Others stated that they were already aware of these issues, either from their own experiences or from recent attention to such issues:

It wasn’t like a shock for me to hear this, because there’s been lots of literature on this and I attend the IHI [Institute for Healthcare Improvement] annual international conference every year. (Manager, male).

Relatedly, many respondents were quick to state that poor workplace behaviour is far from universal and to comment on examples of positive workplace culture:

I had two of them come back to me on the Monday saying that they feel quite protected where we are. … I think it may be a good thing to look at what’s working well in the teams that are functioning at that respectful level and so that we can model some of that. (Nurse Manager, male).

Some respondents emphasised that the need for awareness was highest in groups who are either more likely to be perpetrators (directly or indirectly), or who have most power to effect change:

I think it was quite I guess comforting to see that while I was finding it confronting that it's good for the top brass to be aware of this kind of stuff as well. (Registered Medical Officer, male).

Many respondents spoke at length of how the play increased their insight into the issues it raised:

I think as well we can probably mostly empathise with their situation that the vascular surgeon is having an insanely stressful experience and therefore snaps, I mean it’s normal for people to lash out when they’re that under pressure, and that’s never going to disappear, but there’s a difference between individual incidents of lashing out and a culture of hierarchy in grinding down the people below you. (Nurse, female).

I don’t think after seeing that play will change the way that I interact with especially my seniors … but I think it makes me hyperaware of how I am around other people, like I’ve always had a really huge issue with the way that doctors treat nurses …like being hyperaware of that hierarchical kind of development and not abiding by it, but then again not speaking up to my seniors it might be doing something different to what I think is wrong. (role not supplied, female).

As a result, most respondents stated that they would recommend that colleagues and friends and family should see the play:

I thought it would be helpful for my family to be able to watch this to give them some kind of experience of what it's like. (Human Resources, female)

I’d love to get it out to more staff and encourage them to see it. I know that that’s not practical anymore …There’s no short movie, YouTube or anything on it is there? (Manager, female).

### Intentions to change

We identified sub-themes in this theme: (1) ‘practical intentions for change’; (2) ‘scepticism about change’; (3) ‘management needing to change’; (4) ‘optimism about change’; and (5) ‘performing arts and change.’

A number of respondents stated that the play had prompted them to *consider practical changes* that they could implement immediately as individuals. These included addressing their own symptoms of burnout:

I think my health was only getting worse between seeing the play and now and it was clear that it was unsustainable and something needed to change, so that’s what prompted me to make a plan. (Allied Health, female).

Respondents also reported having increased awareness of others’ burnout; and addressing potential burnout in others by being more available to junior staff and colleagues and by offering to cover shifts where that would be helpful.

Saying is there anything outside of work or do you need to—do you need me to take an afterhours shift for you… in thinking not just oh I've finally got days off; just thinking how does this balance with other people and can I be a bit altruistic with supporting my colleagues. I think it was the main thing that I've done since seeing the play. (Registered Medical Officer, male).

The heightened awareness of the impacts of poor behaviour created by the play motivated some participants to try to prevent such behaviour in themselves:

I've said to the NUM [Nurse Unit Manager] if you see me being rude can you pull me up on it? Because that's the person I don't want to become … it's hard when you're exhausted to step up. (Junior Medical Officer, male).

Other practical intentions included being ‘hyperaware’ of the needs of others, and particularly being more mindful of gender issues specifically; and intentions to explicitly model zero tolerance for workplace misconduct by naming or confronting perpetrators.

Several participants commented on how the play increased the perspective taking that they considered necessary to achieve change:

For me, the thing that will make the biggest change is when we can help people to stand in the shoes of the person – every person in a matter and think about from their perspective for a moment. (Human Resources, female).

Some of the male participants commented that their sensitivity in relation to female colleagues had increased. The play also motivated one participant to directly confront poor behaviour:

After the play, well I had a feedback form that I hadn't filled in for my last term.. and I thought, you know what? I'm actually going to say who the people were that had these certain actions. Because I was like, you know what? We shouldn't have to put up with this. (Registrar, female).

This participant’s action, however, did not achieve a constructive outcome:

They read it and they laughed. … I said, you asked for feedback, I'm just going to write what I honestly saw and I was like okay, I get laughed at when I put that in to work. (Registrar, female).

This experience perhaps reflected the views of many respondents who were *sceptical that culture change* could occur. In part, this was because some perceived *change to require support from high levels of management*:

I think we have heard quite a lot from junior people that they do like the validation but they also say why are we in the audience, why aren’t our managers? They are the ones that should be here and yeah and where is the change going to come from. (Nurse Unit Manager, female).

However many respondents were *optimistic about culture change*, seeing that this was already occurring and might be additionally facilitated by the play:

I think one of the things we need to say about this project is that we started it with the idea that culture doesn’t change overnight. Culture changes because people start to have conversations about what is and isn’t acceptable to them and to draw new boundaries. And that’s exactly what we have in this room tonight. (Physician, female).

I think the play is very graphic, and I think people feel very sad and emotional when they see the play. I think this is our burning platform if you go to change management. (Manager, male).

### Stage 4: interviews (1 year after the play)

Six single interviews were conducted with senior staff with leadership in LHD#2 only (due to COVID disruption). Severe COVID-19 disruption at the time prevented greater participation. Latent thematic analysis was undertaken to answer our third research question, on interview data collected 1 year after the intervention. Analysis identified the theme ‘achieved change’.

Respondents spoke of many spontaneous ways in which their thinking and actions were influenced by the play, especially during interactions with colleagues, and reflected on how seeing the play had facilitated culture change at a local level. Three subthemes were identified: (1) ‘transformative learning’, (2) ‘looking through a new lens’, and (3) ‘increased understanding between groups’.

‘Transformative learning’ describes how recalling the play influenced workplace interactions. One staff member spoke of spontaneously recalling the play “when I’m dealing with workplace conflict”:

[The play is] regularly in my thinking and emotions about our work environment […] it comes up in conversations. (Human resources, female).

Increasing conversation about these issues after the performance and workshop improved staff capacity to address workplace behaviour incidents.

People want to debrief when things occurred, rather than things jumping into a full-blown investigation, having that opportunity to try and nip it in the bud before it turns into something more than what it may be initially. (Senior doctor, male).

‘Looking through a new lens’ refers to the experience of the play enabling enhanced perspective-taking. Participants spoke of how the different viewpoints of the play’s characters, increased the value of perspective taking generally:

Grace Under Pressure puts the different characters on stage in front of you … I think people can identify with themselves in those scenarios. Then there’s that potential to look again through another lens at maybe how I interact with those other roles around me. (Manager, male).

Further, the play translated to “more insights into what being in the shoes of a junior doctor might feel like, with that power imbalance that they experience with seniors’, and understandings such as that “I need to take an equal amount of care for my colleagues as I do for my patients”. (Senior doctor, male).

Relatedly, ‘increased understanding between groups’ refers specifically to a better understanding of the pressures other people face in their roles, and as a result, to improved capacity to reduce “*argy bargy”* and increase dialogue between disciplines and within hierarchies. This not only increased civility, but produced practical improvements in patient safety, for example, including:

All the ancillary staff [get together] in their safety huddles in the morning because these staff are going to be participating in supporting the clinicians when they’re providing care, rolling people over and doing their pressure injury treatments. (Senior Manager, female).

Other concrete changes that had taken place 12 months after the play included establishing a local charter of organisational values; formally integrating these values into existing systems such as performance reviews; monthly recognition of team members living the values well; the creation of individual coaching programs; and establishing a Welfare Officer position. In addition, there had been an increase in complaints, which was interpreted positively:

We’ve actually seen a rise in complaints, which we attribute to increased confidence that there’s somewhere to take those complaints and have them managed. (Human Resources, female).

## Discussion

This mixed methods study assessed the impact of the verbatim theatre play *Grace Under Pressure* on an audience of healthcare staff in 5 hospitals across 3 Local Health Districts, one metropolitan, one regional and one remote. The play, followed by tailored workshops, formed an intervention to improve healthcare workplace culture, by (1) raising awareness (2) facilitating discussion of difficult workplace experiences, and (3) motivating those present to conceive of, and implement, actions to improve culture.

Pre-intervention surveys established that workplace culture needed improvement in the three LHDs where our study was conducted: the majority (60%) of participants were at risk of poor wellbeing (including burnout, fatigue, job strain or suicidal ideation). These results matches the results of two surveys routinely issued by the NSW State Government [37], which is the employer for all public healthcare staff. Employee engagement scores in the 2019 ‘People Matter’ survey found only 65% felt commitment and connection to workplaces. Wellbeing and training scores in the 2018 ‘Your training and wellbeing matters’ survey for junior doctors were very low, indicating high risk of mental illhealth and attrition, with LHD#1 returning the lowest training scores in the State. This confirmed that the intervention was provided in LHDs with considerable need.

Our study showed that *Grace Under Pressure* was very effective in achieving the aims of awareness raising, generating discussion, creation intentions to change and, in one site, facilitating positive change. The close-of-intervention PMC survey strikingly demonstrated the play’s value and impact on a heterogenous audience of healthcare staff: 89% related to the experiences represented in the play, 93% valued the play highly, 92% would recommend that friends, family and the public see it, 89% considered that the play provided insight into poor workplace behaviour and 85% that seeing it made discussing these issues easier.

Thematic analysis of 20 group and single interviews within a month after the play found that the play was very effective in raising awareness – indeed, ‘hyper-awareness’ – of challenging workplace culture issues. Viewing the play’s high fidelity [21] representation of healthcare workplace stressors provided audience members with new insights into how these issues occur and are experienced. The research team has found that video excerpts from the play (CREATE Centre YouTube channel, https://www.youtube.com/@createcentre8294. See eg https://www.youtube.com/watch?v=cW9FGc_uvbs) and used in health professions education, have also achieved such insights [38]. As a result, participants stated intentions to take practical actions to address these issues, including changing their own work habits, being more available to and supportive of their colleagues, and speaking out about poor behaviour. Six interviews conducted in one research site one year following the intervention found that actual improvements in healthcare workplace culture had occurred, and were ascribed in part to the influence of the play. These changes were considered to arise from increased understanding of other colleagues’ workplace pressures, and more capacity to constructively address difficult workplace behaviour.

This study provides evidence that creative interventions can be an effective means of improving healthcare workplace culture. Existing studies of healthcare workplace stressors have repeatedly emphasised that problems such as burnout and mental illhealth cannot be addressed only by treating affected individuals [39]. Instead, a combination of organisational and individual supports are needed to achieve real improvements [40, 41]. *Grace Under Pressure* represented this. The intervention provided an opportunity for whole-of-workplace consideration of the interplay between structural (eg, rostering), cultural (eg teaching by humiliation) and individual (eg perpetrator/struggling employee) factors in difficult workplace experiences.

This study shows that *Grace Under Pressure* was a high impact, time efficient means to activate factors that have been shown to be effective in improving workplace culture. Such factors include: processes that increase staff kindness and compassion to their colleagues, that provide recognition of staff work and needs, that realign working practices to core organisational values [39, 42], and that increase capacity to have constructive ‘courageous conversations’ [13]. Those who saw *Grace Under Pressure* were motivated to think of and undertake practical actions that showed kindness and compassion to colleagues, in part by recognising their colleagues’ personal and work needs. This is in keeping with studies showing that theatre works achieve desired social improvements through small individual actions [43]. A key achievement was that *Grace Under Pressure* prompted some audience members to take steps to prevent poor behaviour *in themselves*. This capacity to *prevent* poor behaviour in potential perpetrators was also found in earlier studies [31, 44]. As a result, one year after the performance, participants described perceived real improvements in workplace culture. They ascribed this specifically to increased ability to conduct difficult conversations (which addressed poor workplace interactions at the time of occurrence), and to increased capacity to enact core organisational values.

This study has shown the importance of longitudinal impact assessment of such interventions. Further longitudinal studies are important to map the multiple pathways through which creative interventions impact healthcare workplaces, and assess the extent and duration of resulting change [45]. We note that it is well known that arts based health interventions achieve outcomes as a result of multiple ‘active ingredients’ [24], making it challenging to assess their impact. Future studies should include complexity in their study design, to capture the multiple and synergistic impacts of creative approaches [46].

In April 2023 PMC accepted yet another commission from local hospitals to perform Grace Under Pressure – in this case, specifically as a means of supporting healthcare workers experiencing a burnout crisis, due to the ongoing impact of COVID-19. Such continuing requests for the play, 6 years after its initial season and 2 years after a national mainstage tour, constitute very strong evidence for the play’s impact and value.

### Limitations

Resourcing constraints, which were uneven across LHDs, limited consistency in recruitment for Stage 1, impacted on sample sizes, and precluded much capacity to make comparisons across settings in this study. Covid-19 disruptions severely limited the participant recruitment at 1 year post intervention.

## Conclusions

### Summary of findings

This study found that the verbatim theatre play *Grace Under Pressure* was highly valued by its healthcare staff audiences because it made visible real workplace conditions and behaviour that contribute substantially to poor staff wellbeing, and provided validation of staff suffering as a result of them. The study found that the play achieved the aims of raising awareness about, and discussion of, poor workplace behaviour and workplace stressors, providing healthcare worker audiences with considerable insight into how burnout and mistreatment arise in healthcare work. The play was successful in motivating members of its audience to take small actions to mitigate these problems, and this resulted in real improvements in workplace culture in the one setting where longitudinal data could be collected despite COVID-19 disruption.

### Implications

Verbatim theatre is a powerful tool in achieving real organisational change. It is likely that other creative arts based methodologies will similarly achieve significant impacts when used to address other complex, multi-faceted organisational challenges in healthcare delivery.

### Supplementary Information

**Supplementary Material 1.**

## Data Availability

The datasets generated and analysed during the current study are not publicly available due to human research ethics approval requirements to maintain confidentiality and privacy and due to intellectual property provisions at the Pam Maclean Centre. They are stored securely in accordance with human research ethics approval requirements and are available from the corresponding author upon reasonable request.

## Abbreviations

LHD: Local Health District, an administrative unit that delivers operational healthcare under the direction of the NSW Government Ministry for Health
NSW: New South Wales, a State in Australia
PMC: Pam McLean Centre, a healthcare communication centre that offers theatre and drama based resources to improve healthcare communication, and is affiliated with the University of Sydney
