# Supplementary material for: Grace Under Pressure: a mixed methods impact assessment of a verbatim theatre intervention to improve healthcare workplace culture
Source: BMC Health Serv Res. 2024 Apr 16;24:474. doi: 10.1186/s12913-024-10961-w (PMC11022423; doi:10.1186/s12913-024-10961-w)
Supplement: Supplementary file 1 — Supplementary Material 1. [file 12913_2024_10961_MOESM1_ESM.docx]

**Supplementary Materials**

Video excerpts from *Grace Under Pressure* can be provide as hyperlinks. They are held at <https://www.youtube.com/@createcentre8294/featured>. Specific hyperlinks can be supplied.
